# Factors Influencing the Onset of Intra- and Post- Operative Complications Following Tooth Exodontia: Retrospective Survey on 1701 Patients

**DOI:** 10.3390/antibiotics8040264

**Published:** 2019-12-13

**Authors:** Erica Vettori, Fulvia Costantinides, Vanessa Nicolin, Roberto Rizzo, Giuseppe Perinetti, Michele Maglione, Roberto Di Lenarda

**Affiliations:** Maxillofacial and Dental Surgical Clinic, Department of Medical, Surgical and Health Sciences, University of Trieste, Piazza dell’Ospitale 1, 34129 Trieste, Italy; F.COSTANTINIDES@fmc.units.it (F.C.); nicolin@units.it (V.N.); R.RIZZO@fmc.units.it (R.R.); M.MAGLIONE@fmc.units.it (M.M.); rdilenarda@units.it (R.D.L.)

**Keywords:** antibiotic therapy, tooth extraction, alveolitis, smoke, wound healing, post-operative complications

## Abstract

Complications after tooth extraction may occur because of several factors correlated to the patient’s medical history, surgical site or type of intervention. The aim of this retrospective cohort study was to evaluate type and frequency of complications after exodontic surgery, its correlation with antibiotic administration and between patient’s related systemic factors. From June 2015 until February 2016 1701 exodontic interventions, for a total of 2322 extracted teeth, were carried out at the Unit of Oral Surgery in Trieste. Descriptive statistic, and backward multiple logistic regressions were performed to identify the variables associated with the presence of post-operative alveolitis or any other post-operative complication. The presence of coagulopathy and smoking habit were related to high risk of post-operative alveolitis (OR = 5.51, *p* = 0.035 and OR = 2.5, *p* = 0.029, respectively). Tooth fracture was found to be correlated with higher probability of post-operative alveolitis (*p* = 0.001) and concomitant chemotherapy put at a higher risk post-operative complications, including alveolitis (OR = 29.5, *p* = 0.018). According to the present results, antibiotic consumption did not seem to reduce the incidence of post-operative infective complications (alveolitis). A careful analysis of medical history, the adequate surgical technique, and the correct instructions in post-surgical behavior, prevent the insurgence of intra and post-operative complications.

## 1. Introduction

Tooth extraction is one of the oldest and most common oral surgical procedures that dentists and oral surgeons carry out.

Indications for tooth extraction include advanced periodontal disease, abscess or phlegmon, non-restorable caries, residual roots, tooth fracture, failed endodontic treatments with the persistence of periapical granulomas or cysts and third-molar impaction [1]. Extraction may also be part of an orthodontic or prosthodontic treatment plan. After surgical interventions, various types of complications may arise such as alveolitis, oedema and lockjaw, bone spicules, post-operatory bleeding, and paresthesia [2,3].

Complications are multifactorial and are correlated either to the patient’s health status or habits or to systemic and local factors. First, patients’ lifespan is increasing and dentists visit a growing number of medically compromised patients; secondarily, some of the most common complications of surgical procedures may be influenced by patients’ medical history [4,5].

Many clinical conditions may influence wound healing such as diabetes mellitus (DM) where healing impairment is characterized by a number of local cytokine and cellular abnormalities such as reduced angiogenesis and decreased collagen synthesis [6]; Human Immunodeficiency virus- infection (HIV) because of the progressive destruction of the immune system [7]; chronic obstructive pulmonary disease (COPD) because of reduced oxygen supply, Cushing’s Syndrome, anemia, and malnutrition [8]. Also concomitant oncologic treatment (radio- or chemotherapy), hepatic disease, kidney failure, thyroid disease as well as immuno-suppression and prolonged corticosteroid therapy negatively influence wound healing [9,10].

The healing process culminates with the deposition and remodeling of the woven bone, which is promoted by the blood filling of post-extraction sockets. Factors affecting clot formation include smoking, which may, in general, have an adverse effect on healing of the extraction wound. In vitro studies showed that nicotine negatively affects osteoblasts, inhibits fibroblast growth, production of collagen and fibronectin, while promoting collagen breakdown [11].

Drug consumption may also influence the onset of intra and/or postoperative complications as in the case of bleeding due to anticoagulant or antiplatelet therapies as well as in the case of platelet disorders associated with liver disease or bleeding caused by hypertension [12,13].

Also, complications are more likely to occur in the case of complicated/multiple extractions or peculiar anatomic conditions: these could depend on the site of surgery. For example, maxillary tuberosity fracture or oro-antral fistulae when root apices of maxillary teeth are connected with the maxillary sinus [14] or, in the mandible, the proximity of the inferior alveolar nerve. Post-operative complications, including swelling, infection of the surgical site, and post-operative bleeding, may also be related to surgical intervention since impacted/decayed/fractured teeth generally require a more invasive procedure [15].

The administration of an antibiotic therapy before or after extractions to prevent complications after tooth exodontia is still controversial. There is no agreement in the literature regarding the effectiveness and appropriation of antibiotic prescription on preventing surgical site infections such as alveolitis [16,17,18].

The primary objective of this retrospective study was to evaluate the correlation between the administration of prophylactic or post-operative antibiotic therapy with the onset of complications after single or multiple dental extractions. The secondary objective of our research was to possibly correlate the onset of complications with pre- or post-operative features with health status, habits or medical history of the enrolled patients. Alveolitis was considered as the main outcome among the complications in the analysis.

## 2. Material and Methods

The present retrospective study was conducted at the Unit of Oral Surgery of the School of Dental Sciences (University of Trieste, Trieste, Italy) between June 2015 and February 2016.

The study was conducted in accordance with the Declaration of Helsinki, and the protocol was approved by the Research Ethics Committee of the University of Trieste, Trieste, Italy (n.73/2016) and all participants signed a privacy informed agreement as per hospital protocol. The study design followed the STROBE checklist for cohort studies [19].

All patients who underwent single or multiple tooth extractions in the selected period of time were included in the present study.

Exclusion criteria were patients subjected to periodontal surgery or major oral surgery, patients without specification of which antibiotic was prescribed after extraction.

Data were collected following examination of the patients’ record by an oral surgeon (E.V.).

For each patient demographic and admission data were collected, that is, age, sex (male/female), year of intervention (2015/2016), month (June/July/August/September/October/November/December/January/February) and season (winter/spring/summer/autumn); regarding medical history presence of diabetes mellitus (both type-1 and type-2), osteoporosis, anemia, coagulopathies, hypertension, heart failure, kidney failure, autoimmune disease, thyroid disease, chronic obstructive pulmonary disease (COPD), liver cirrhosis, Hepatitis B virus-infection (HBV), Hepatitis C virus-infection (HCV), HIV-infection and smoking habit were reported. Also we considered if the patient had had radiotherapy performed in the head and neck region and if drug consumption was present, namely, antiplatelet and anticoagulant drugs, steroids, intravenous and oral bisphosphonates, immunosuppressive drugs, colchicine, concomitant chemotherapy and polypharmacological treatment.

Moreover, it was reported if antibiotic therapy had been prescribed previously or after surgery (type of antibiotic, dose and timing were collected).

Also the onset of intra-operative complications such as cortical or maxillary tuberosity fracture, bleeding and oroantral fistula were evaluated.

Finally, the onset of post-operative complications, in particular, dry socket, oedema and lockjaw, bone spicule, post operatory bleeding and paresthesia were recorded.

## 3. Data Management

The SPSS software (SPSS^®^ Inc., Chicago, IL, USA) was used to perform the data analysis. The presence of post-operative complications, including alveolitis, were considered as the distinct main outcome and used as the dependent variables in all the analyses.

Descriptive statistics (frequencies or mean ± SD) were reported for the following records: (1) medical history variables (as yes, no): diabetes, osteoporosis, anemia, coagulopathy, hypertension, heart failure, kidney failure, autoimmune disease, thyroid disease, COPD, Cushing’s Syndrome, cirrhosis, HBV, HCV, HIV, antiplatelet therapy, anticoagulant therapy, steroids, intravenous bisphosphonates, oral bisphosphonates immunosuppressive therapy, colchicine, chemotherapy, head and neck radiotherapy, other drugs, smoker; (2) surgical treatment variables: reason for extraction (periodontal disease, caries of non-restorable tooth or endodontic treatment failure, dysodontiasis, tooth fracture, abscess or phlegmon), number of teeth removed, type of element removed (primary tooth, permanent tooth, residual root, impacted tooth), site of intervention (posterior maxillae, anterior maxillae, posterior mandible, anterior mandible), surgical technique (simple, surgical extraction; surgical extraction if flap and ostectomy were performed was considered), anesthesia (mepivacain with adrenaline 1:100,000, mepivacain without adrenaline), execution of osteoplastic after tooth removal (yes, no), type of stitches used (non-absorbable sutures [silk], absorbable sutures [Vicryl^®^], not used), consumption of antibiotics before the intervention (no, Amoxicillin and Clavulanic acid, Amoxicillin, Macrolides, Quinolones), the prescription of drugs after the surgery (no, Amoxicillin and Clavulanic acid, Amoxicillin, Macrolides, Quinolones), prescription, after surgery of non-steroidal anti-inflammatory drugs (NSAIDs) or corticosteroids (as yes, no).

For each explanatory variable, a chi-squared test was performed to assess separately the significance of the differences in the incidence of alveolitis or any post-operative complication (not shown). The explanatory variables were subsequently included, each in a multivariate analysis when the corresponding p-value of the univariate analyses was lower than 0.40. In particular, backward multiple logistic regressions were performed to identify the variables that were associated with the presence of a post-operative alveolitis or any post-operative complication. The cut-off levels of significance used were 0.05 and 0.10 for entry and removal, respectively. A *p*-value <0.05 was considered as being statistically significant.

## 4. Results

A total of 1701 exodontic interventions for a total number of 2322 extracted teeth (mean 1.37 extracted teeth per intervention) was performed in the selected patients. Of the patients 876 were males and 845 females, with a mean age of 55.3 ± 19.9 years, 41 interventions were performed in winter, 534 in spring, 752 in summer, and 374 in autumn.

Descriptive frequencies for the whole sample are summarized in Table 1 and Table 2.

Concerning medical history, more than one third of patients was affected by hypertension and more than 10% by diabetes. Regarding autoimmune diseases, the most frequently encountered pathologies were psoriasis, rheumatoid arthritis, pemphigus, pemphigoid, Sjögren’s syndrome, erythematous systemic lupus.

With regard to infectious diseases, the most commonly found was HCV (5%), although a prevalence of HIV and HBV were encountered too.

In the sample only 661 patients (38.86%) were not taking any medications, whereas more than 60% patients were found to be undergoing treatment with one or more chronic therapies. Detailed results are displayed in Table 1. Antiplatelet therapy was the most frequently assumed but a significant percentage of patients was found to be on anticoagulants such as acenocumarol or warfarin (8%).

Almost 30% of patients were smokers.

Data regarding the surgical procedure are reported in Table 2.

Reasons for extraction were periodontitis, caries, dysodontiasis, fracture and abscess or phlegmon, almost equally distributed between the maxillary and the mandibular region. Few patients underwent exodontic surgery in both jaws at the same time. In 51% of cases a permanent tooth was extracted; in 33.45% a residual root, in almost 10% a fully or partially impacted tooth. In 15.70% of extractions a flap was performed to permit extraction operations and to allow primary intention healing, whereas osteoplasty during intervention was necessary in 15.64% of cases. The majority of patients received local anesthesia with adrenaline (80.31%), and in almost the whole sample (99.89%) sutures were placed after extraction.

In 10.47% of cases the patient had started an antibiotic therapy before the intervention. After the intervention, the surgeon prescribed antibiotic therapy to 9.23% of patients, steroids to 0.24% of patients, NSAIDs to 3% of patients. Detailed data on prescriptions can be found in Table 2.

Results of the multivariate analysis are summarized in Table 3.

Each explanatory variable was considered in the multiple backward regressions to estimate the association with the presence of post-operative alveolitis alone and of all the other post-operative complications (dry socket, oedema and lockjaw, bone spicule, post operatory bleeding and paresthesia including alveolitis) considered as a whole; Model 1 shows the variables associated with post-operative alveolitis considered alone; Model 2 summarizes dependent variables associated with the presence of post-operative complications (oedema and lockjaw, bone spicule, post operatory bleeding, paresthesia) including alveolitis.

The presence of coagulopathy and smoking habit were related to a high risk of post-operative alveolitis (OR = 5.51, *p* = 0.035 and OR = 2.5, *p* = 0.029 respectively).

Reason for extraction and above all root fracture, were found to be correlated with a higher probability of post-operative alveolitis (*p* = 0.001).

Patients who were undergoing a concomitant chemotherapy were associated with a higher risk of developing post-operative complications (OR =29.05 CI = 1.78–487.7, *p* =0.018), as well as post- operative osteoplasty execution (OR = 2.40, CI = 1.14–5.15, *p* =0.020); similarly, patient who were under polypharmacological treatment (OR = 1.9, CI = 1.01–3.70, *p* = 0.040) were more likely to develop complications.

Among motivation for tooth extraction only dental fracture was statistically significant (*p* = 0.000) for the onset of post-operative complications.

## 5. Discussion

The most common complication of tooth extraction is dry socket; a distinct etiology of this complication is not defined, but generally the increase of fibrinolytic activities is considered as the main etiologic factor that causes a dissolution of the blood clot. Momeni H. et al. [20] demonstrated that when anti-fibrinolytic agents were placed at the site of tooth extraction, the incidence of dry socket was reduced.

Moreover, several studies demonstrated the correlation between dry socket and smoking habit [21,22]; cigarette smoking reduces the post-operative socket filling with blood, so wound healing is slowed down [23]. Furthermore, the presence of nicotine, which is a cytotoxic substance [24], interferes with the expression of a number of genes which play an essential role in the formation of new vessels and in bone healing, leading to an increased catabolic response that may be unfavorable to new bone formation, negatively affecting wound healing [25].

In this retrospective study the percentage of people who smoked was 29.7% but if we consider only patients who incurred post-surgical complications, the percentage of smokers was considerably higher (52%).

Nevertheless, some authors state that dry socket is increased after traumatic tooth extractions (e.g., surgical extractions) but not specifically in smokers [26].

According to results of the multiple backward logistic regressions presented in this study, the presence of coagulopathy, smoking habit and reason for extraction, in particular periodontitis, caries, fracture, and abscess were associated with development of post-operative complications and alveolitis.

In this study almost all surgical sites had been sutured, and the percentage of post-operative complications was very low. Literature demonstrates that after a tooth extraction, the socket heals with the formation of a stable clot, followed by migration of epithelium onto the clot and remodeling of the alveolar bone; for this reason the application of sutures over the wound may stabilize the clot and help socket healing [27].

Literature reports a percentage of post-surgical complication after oral surgery in head and neck cancer patients that ranges from 10% to 45% [28]; in our sample the percentage of all post-surgical complications was only 3.17%. This finding could be explained because of the stability of the coat ensured by stitches. Svensson R et al. showed that the use of local hemostatic agents, sutures, and tranexamic acid reduced the risk of postoperative bleeding after tooth removal in patients assuming anticoagulant therapy [29,30].

Anaand KP et al. [31] reported the efficacy of tranexamic acid application after tooth extraction to promote wound healing for prevention of post-surgical complications such as alveolitis, this conforms with the correlation between the presence of coagulopathy and the development of alveolitis presented in this study. Coagulation disorders impede the formation of a stable blood clot and considering that the disintegration of the initial blood clot formed inside the alveolar socket causes the failure of socket healing, dry socket occurs.

Model 2 of the multiple backward logistic regression reports a correlation between polypharmacological treatment, concomitant chemotherapy, and osteoplasty execution and occurrence of post-operative complications (including alveolitis).

Bleeding after dental extractions is sometimes affected by pharmacologic therapies like antiplatelet/anticoagulant/anti-hypertensive drugs; literature reports show that dental extraction can be performed safely in patients treated with antiplatelet therapy [32] without withdrawal therapy because the suspension of antiplatelet therapy puts the patient at a higher risk of cardiovascular events [33,34] than the intervention itself. In this study patients on antiplatelet or anticoagulant drugs or suffering from hypertension were 18.1%, 7.9%, and 33.8%, respectively, and the reported percentage of hemorrhage was 0.4%. For every patient that assumed anticoagulant therapy the International Normalized Ratio (INR) was measured before performing the extraction and if it was higher than 3.5 the intervention was postponed. None of the patients interrupted antiplatelet or anticoagulant therapy before the surgery and the results confirm that dental extractions can be carried out safely in these patients [35].

Tarakji B et al. [30] in their systematic review made a list of risk factors associated with the development of alveolitis alone after exodontia; these were surgical trauma, dose of anesthesia, site and number of extractions, age, sex, medical history and systemic disorders, operator experience and difficulty of the surgery, antibiotics use prior to surgery, previous infection of the surgical site in addition to oral contraceptives, menstrual cycle and immediate post-extraction socket irrigation with normal saline solution. In this study the only factors associated with development of alveolitis were: “presence of coagulopathy”, “smoking habit”, “surgical trauma” (when motivation for extraction was tooth fracture) and “previous odontogenic abscess” [36].

It is easily deducible that patients undergoing tooth extractions during chemotherapy are more predisposed to develop complications during socket healing as chemotherapy-induced adverse reactions are not uncommon [37].

According to our backward multiple logistic regression we found that concomitant chemotherapy is likely to have an enormous significance in the insurgence of post-operative complications (including alveolitis). If we consider that number of patients is exiguous, this evidence is strengthened.

Diabetes is another condition known to expose patients at a high risk of slow healing and wound infection following a surgical intervention [38]. However, it remains unclear whether those who undergo tooth extractions are more likely to develop surgical infection as compared to non-diabetic patients [39,40]. In the present study diabetes does not seem to interfere with the post-operative course.

Anyway, complications after oral surgery may occur not only because of medical history or pharmacological treatments; in fact, there are also complications due to the surgical site, for example wisdom tooth extraction and its proximity with the inferior alveolar nerve.

To prevent iatrogenic complications such as paresthesia of the inferior alveolar nerve, root fractures or critical bleeding during invasive surgical procedures, a useful diagnostic tool is needed.

Pathak S et al. [2] suggested the execution of a computed tomography (CT) or a Cone Beam Computed Tomography (CBCT) for a more precise evaluation of the relationship between the third molar and noble structures like the inferior alveolar nerve. Indeed, a panoramic radiography is not always reliable [41]. Although this may be of great interest for further investigations, in this study the number of post-operative paresthesiae was too exiguous to evaluate the effectiveness of the prescription of second level imaging examinations for the pre-operatory evaluation of surgical sites.

Following the results of this survey, antibiotic therapy administrated before tooth extraction does not seem to influence the insurgence of post-exodontic infections. The multivariate analysis showed that antibiotic administration before oral surgery does not reduce the incidence of post- operative alveolitis (*p*-value > 0.05).

Literature does not give strict guidelines concerning antibiotic administration after exodontia for the prevention of post-operative infection, and many studies disagree with the effectiveness of antibiotic treatment before and after dental procedures such as tooth extraction.

Arteagoitia I et al. [42] in their double-blind placebo-controlled clinical trial, did not find statistical significance in the insurgence of post-operative infections in patients treated with antibiotic therapy after surgical extraction of completely bone-impacted third molars versus not treated patients. Also, Luaces-Rey R et al. [43] found out that administration of amoxicillin after exodontia is not justified. They demonstrated a non-statistically significant difference between a first group of patients treated with antibiotics after surgery and a non-treated group.

However, it has been well demonstrated that during dental extractions bacteremia occurs. Moreno-Drada JA et al. [44] in their systematic review and meta-analysis found that in 100% of patients who underwent dental extraction bacteremia was observed, whereas the percentage was lower during other dental procedures such as endodontic treatment or implant placement. The incidence of bacteremia decreased when antibiotic prophylaxis was administered and, according to this study, it could also reduce post-operative occurrence of infections.

In recent times the problem of drug-resistant bacteria is growing so the medical community is trying to regulate the use of antibiotics, also after dental procedures. From many studies, it can be deduced that antibiotic therapy in healthy people does not reduce the incidence of post-operative complications [45], so prescriptions should be aimed at reducing the risk of antibiotic resistance occurrence.

Controversially, some authors affirm that antibiotic therapy after third molar surgery is useful in post-operative recovery; in particular, López-Cedrún et al. [46] in their double blind controlled study compared two regimens of antibiotic therapy in patients undergoing third molar surgery, before and after surgery. The results showed that amoxicillin administration after surgery had a statistically significant beneficial effect in post-operative recovery.

At present, administration of antibiotics to prevent post-extraction complications is controversial; the decision on whether to prescribe or not antibiotics after dental extraction is based on a risk assessment considering that the factors involved in the risk-benefit evaluation may be approached differently by experts. Therefore, practitioners need to carefully discuss the potential benefits and harms of antibiotic prescription with their patients.

## 6. Conclusions

When exodontic surgery is well conducted and surgical trauma is minimized with an appropriate surgical technique, the stability of the coat is facilitated by stitches application, patients who use anticoagulant drugs are therapeutically anti-coagulated (International Normalized Ratio [INR] from 2 to 3) and when patients follow post-operative instructions supplied such as abstinence from smoking and avoidance of mouth rinses, complications are preventable. From our results administration of antibiotics before or after dental extraction, is not effective in preventing post- surgical complications.

## Figures and Tables

**Table 1:** Frequencies of the explanatory variable considered in the study (*n* = 1.701).

Explanatory Variable	Count (%)
Diabetes mellitus	212 (12.46%)
Osteoporosis	101 (5.94%)
Anemia	85 (5.00%)
Coagulopathies	24 (1.41%)
Hypertension	576 (33.86%)
Heart failure	27 (1.59%)
Kidney failure	58 (3.41%)
Autoimmune disease	62 (3.64%)
Thyroid disease	117 (6.88%)
COPD	74 (4.35%)
Liver cirrhosis	25 (1.47%)
HBV	28 (1.65%)
HCV	89 (5.23%)
HIV	3 (0.18%)
Smoke	505 (29.69%)
Antiplatelet therapy	308 (18.11%)
Anticoagulant therapy	134 (7.88%)
Steroids	29 (1.70%)
Intravenous Bisphosphonates	4 (0.24%)
Oral Bisphosphonates	37 (2.18%)
Immunosuppressive therapy	24 (1.41%)
Colchicine	1 (0.06%)
Chemotherapy	2 (0.12%)
Head and neck radiotherapy	15 (0.88%)
Polypharmacological treatment	975 (57.32%)

COPD: chronic obstructive pulmonary disease; HBV: Hepatitis B virus-infection; HCV: Hepatitis C virus-infection; HIV: HIV-infection.

**Table 2:** Frequencies of the explanatory variable considered in the study (*n* = 1.701).

Explanatory Variable	Count (%)
Reason for extraction	
Periodontitis	532 (31.29%)
Caries	964 (56.67%)
Dysodontiasis	191 (11.23%)
Fracture	46 (2.70%)
Abscess	70 (4.12%)
Type of element	
Permanent Tooth	871 (51.21%)
Residual Root	569 (33.45%)
Full or Partial Impacted Tooth	165 (9.70%)
Deciduous Tooth	2 (0.12%)
Miscellaneous	94 (5.53%)
Site	
Anterior Maxilla	171 (10.05%)
Posterior Maxilla	624 (36.68%)
Anterior Mandible	138 (8.11%)
Posterior Mandible	645 (37.92%)
Miscellaneous	123 (7.23%)
Surgical technique	267 (15.70%)
Anesthesia	1366 (80.31%)
Osteoplasty	266 (15.64%)
Suture	
Silk	1514 (89.01%)
Vicryl^®^	185 (10.88%)
None	2 (0.12%)
Antibiotic before surgery	
Amoxicillin and Clavulanic acid	99 (5.82%)
Amoxicillin	69 (4.06%)
Macrolides	7 (0.41%)
Quinolones	2 (0.12%)
Amoxicillin and Metronidazole	1 (0.06%)
Antibiotic after surgery	
Amoxicillin and Clavulanic acid	86 (5.06%)
Amoxicillin	62 (3.64%)
Macrolides	8 (0.47%)
Quinolones	1 (0.06%)
Steroids after surgery	4 (0.24%)
NSAIDs after surgery	51 (3.00%)

No., number of cases. Negative cases for each explanatory variables are not reported.

**Table 3:** Results of the multiple backward logistic regressions for estimates of association of post- operative alveolitis or post-operative complication (including alveolitis) with each explanatory variable (*n* = 1.701).

Variable	OR	95% CI	Significance
**Model 1: Dependent Variable, Post-Operative Alveolitis**			
Coagulopathy			
No	1		
Yes	5.51	1.13–26.82	*p* = 0.035; **S**
Smokers			
No	1		
Yes	2.5	1.10–5.69	*p* = 0.029; **S**
Extraction indication			
Periodontitis	1		
Caries	6.47	0.85–49.20	*p* = 0.071; **S**
Dysodontiasis	4.86	0.44–54.05	*p* = 0.199; NS
Fracture	46.13	5.01–425.18	*p* = 0.001; **S**
Abscess	13.68	1.22–153.63	*p* = 0.034; **S**
**Model 2: Dependent Variable, Post-Operative Complications (Including Alveolitis)**			
Chemotherapy			
No	1		
Yes	29.5	1.78–487.7	*p* = 0.018; **S**
Polypharmacological treatment			
No	1		
Yes	1.9	1.01–3.70	*p* = 0.04; **S**
Osteoplasty			
No	1		
Yes	2.4	1.14–5.15	*p* = 0.020; **S**
Extraction indication			
Periodontitis	1		
Caries	2.09	0.85–5.11	*p* = 0.106; NS
Dysodontiasis	2.55	0.72–9.01	*p* = 0.146; NS
Fracture	11.3	3.59–35.92	*p* = 0.000; **S**
Abscess	2.15	0.42–11.07	*p* = 0.362; NS

**OR**, adjusted odds ratio; 95% CI, 95% confidence interval of the OR. Reference category for the dependent variables defined as no post-operative alveolitis or no post-operative complication (including alveolitis); **S**, statistical significant; **NS**, not statistically significant.
